# Physiological and Biochemical Responses of *Ageratum conyzoides*, *Oryza sativa* f. *spontanea* (Weedy Rice) and *Cyperus iria* to *Parthenium hysterophorus* Methanol Extract

**DOI:** 10.3390/plants10061205

**Published:** 2021-06-14

**Authors:** Mst. Motmainna, Abdul Shukor Juraimi, Md. Kamal Uddin, Norhayu Binti Asib, A. K. M. Mominul Islam, Muhammad Saiful Ahmad-Hamdani, Zulkarami Berahim, Mahmudul Hasan

**Affiliations:** 1 Department of Crop Science, Faculty of Agriculture, Universiti Putra Malaysia, Serdang 43400, Selangor, Malaysia; gs51794@student.upm.edu.my (M.M.); s_ahmad@upm.edu.my (M.S.A.-H.); gs53801@student.upm.edu.my (M.H.); 2 Department of Land Management, Faculty of Agriculture, Universiti Putra Malaysia, Serdang 43400, Selangor, Malaysia; mkuddin@upm.edu.my; 3 Department of Plant Protection, Faculty of Agriculture, Universiti Putra Malaysia, Serdang 43400, Selangor, Malaysia; norhayuasib@upm.edu.my; 4 Department of Agronomy, Faculty of Agriculture, Bangladesh Agricultural University, Mymensingh 2202, Bangladesh; akmmominulislam@bau.edu.bd; 5 Laboratory of Climate-Smart Food Crop Production, Institute of Tropical Agriculture and Food Security, Universiti Putra Malaysia, Serdang 43400, Selangor, Malaysia; zulkerami@upm.edu.my

**Keywords:** *P. hysterophorus*, photosynthesis, chlorophyll, antioxidant enzymes, malondialdehyde, proline, *A. conyzoides*

## Abstract

The current study was designed to investigate the effect of *Parthenium hysterophorus* L. methanol extract on *Ageratum conyzoides* L., *Oryza sativa* f. *spontanea* (weedy rice) and *Cyperus iria* L. in glasshouse condition. Here, *Parthenium hysterophorus* methanol extract at 20, 40, and 60 g L^−1^ concentrations was applied on the test species to examine their physiological and biochemical responses at 6, 24, 48 and 72 h after spraying (HAS). The phytotoxicity of *P. hysterophorus* was strong on *A. conyzoides* compared to weedy rice and *Cyperus iria* at different concentrations and exposure times. There was a reduction in photosynthesis rate, stomatal conductance, transpiration, chlorophyll content and carotenoid content when plants were treated with *P. hysterophorus* extract concentrations. Exposure to *P. hysterophorus* (60 g L^−1^) at 24 HAS increased malondialdehyde (MDA) and proline content by 152% and 130%, respectively, in *A. conyzoides* compared with control. The activities of antioxidant enzymes (superoxide dismutase (SOD), catalase (CAT) and peroxidase (POD)) were also increased in the presence of *P. hysterophorus* extract. Present findings confirm that the methanol extract of *P. hysterophorus* can disrupt the physiological and biochemical mechanism of target weeds and could be used as an alternative to chemical herbicides.

## 1. Introduction

Weeds sometimes compete with crops for space, nutrients, water, and light, resulting in growth reduction and yield loss of cultivated crops. *Ageratum conyzoides* (L.) L., *Oryza sativa* f. *spontane* Roshev. (weedy rice) and *Cyperus iria* L. are common weeds in the cultivated lands. *Ageratum conyzoides* could be considered an aggressive weed and can infest cultivated agricultural land and cause a severe yield loss by interfering growth and development of different crops [1]. Weedy rice possesses similar characteristics to cultivated rice and has a long unsettled problem in rice granaries [2]. It has been reported that weedy rice caused yield loss in rice fields from 16% to 74% in Asia [3,4,5]. *Cyperus iria* is an annual herbaceous sedge and responsible for reducing crop yield, particularly in rice and caused a 64% reduction in rice yield [6].

*Parthenium hysterophorus* is considered an invasive weed, which belongs to the Asteraceae family. It creates severe problems in human health, crop production and animal husbandry [7]. High germination rate, prolific nature and resistance to chemical herbicide are the major challenges to control this noxious weed. The allelopathic potential of certain weed and crop species can influence the growth and distribution of associated weed species and the yield of desired plants. Moreover, allelopathy has been employed successfully in biocontrol programs focusing on the control of problematic weeds and plant diseases [8]. Previous reports have shown that *P. hysterophorus* has the allelopathic potential [8,9,10], which could be introduced in the weed management program.

Various physiological and biochemical responses, including chlorosis, lipid peroxidation and antioxidant responses, can occur in plants as a consequence of herbicide exposure [11]. Chemical stress is abiotic stress that obstructs a plant’s growth and, when applied in high concentrations, could be lethal. Abiotic and biotic conditions dictate the physiological functions and stability of the plant. However, various abiotic stresses lead to the overproduction of reactive oxygen species (ROS) in plants, which are highly reactive and toxic, causing damage to proteins, lipids, carbohydrates and DNA, which ultimately results in oxidative stress [12]. Chlorophylls are the fundamental pigments that play a key role in photosynthesis. Carotenoids play an important photo-protective role by scavenging ROS and suppressing lipid peroxidation [13]. Differences in plant leaf chlorophyll and carotenoid content reflect their resistance levels and photosynthetic capacities [14]. Under oxidative stress, plants respond by increasing antioxidant defenses, particularly enzymes such as superoxide dismutase (SOD), peroxidase (POD) and catalase (CAT) [15]. SOD happens in several cell compartments and catalyzes the dismutation of the superoxide anion into hydrogen peroxide (H_2_O_2_) and molecular oxygen (O_2_) [16]. Hydrogen peroxide, in turn, is removed by several other antioxidant enzymes, such as CAT and POD [17]. Malondialdehyde (MDA) is a metabolic product of lipid peroxidation and a widely accepted indicator of oxidative damage in plants [18]. Lipid peroxidation affects the physiological process of the cell [19].

Many studies are available on allelopathy, but not much is known about its mechanism of activity. This study aims to determine physiological and biochemical changes resulting from the implementation of *P. hysterophorus* extract concentration on *A. conyzoides*, weedy rice and *C. iria*. An understanding of the physiological and biochemical phenomenon of allelopathy will no doubt explicate the mechanism of action and the possible target site. Thus an investigation into the allelopathic potential of *P. hysterophorus* was conducted.

## 2. Results

### 2.1. Effect of P. hysterophorus Extract on Chlorophyll-a Content of the Test Plant Species

Foliar spray of *P. hysterophorus* extract had a significant effect on chlorophyll-*a* content of *A. conyzoides*, weedy rice and *C. iria* at 6, 24, 48 and 72 h after spray (HAS) (Table 1). Chlorophyll-a content showed a decline from 12.50 to 31.98%, 30.01 to 74.32%, 29.78 to 73.53% and 31.10 to 74.09% in *A. conyzoides* compared to control (0 g L^−1^) at 6, 24, 48, and 72 HAS, respectively by foliar application of *P. hysterophorus* at different concentrations. Simultaneously, only a 36% decline was observed in weedy rice and *C. iria* after 24 h at the highest concentration (60 g L^−1^) of *P. hysterophorus* extract.

### 2.2. Effect of P. hysterophorus Extract on Chlorophyll-b Content of the Test Plant Species

The chlorophyll-*b* content was significantly affected by the foliar spray of *P. hysterophorus* extract, but no significant effect was observed at 20 g L^−1^ between the HAS in *A. conyzoides* (Table 1). The foliar spray concentration, however, reduced the chlorophyll-*b* content of *A. conyzoides* at 24 HAS from 25.55 to 67.50% at the lowest (20 g L^−1^) to highest (60 g L^−1^) concentration compared to the control (0 g L^−1^). The result also exhibited that the response of weedy rice was not significantly different in the lowest (20 g L^−1^) concentration but differed from the highest (60 g L^−1^) concentration and control (0 g L^−1^) at different hours of spray. *C. iria* showed a 34.88% decreased in chlorophyll-*b* content compared to control (0 g L^−1^) at 24 HAS at the highest (60 g L^−1^) concentration of *P. hysterophorus* extract. *A. conyzoides* showed the highest reduction in chlorophyll-*b* content compared to other test plants and this was a relatively much greater impact on the green pigment.

### 2.3. Effect of P. hysterophorus Extract on Total Chlorophyll Content of the Test Plant Species

Total chlorophyll content was significantly affected by the interaction of hours after foliar spray and *P. hysterophorus* extract concentration. The total chlorophyll content of *A. conyzoides* was declined 12.79%, 19.67%, and 31.31% at 6 HAS, while 22.78%, 45.31% and 72.45% reduction was obtained at 24 HAS for the foliar application of 20 g L^−1^, 40 g L^−1^ and 60 g L^−1^, respectively compared to control (0 g L^−1^) (Table 2). Around 34% reduction occurred in the total chlorophyll content of weedy rice, and *C. iria* at 72 HAS by applying the highest (60 g L^−1^) amount of *P. hysterophorus* extract. Weedy rice and *C. iria* maintained relatively higher chlorophyll content than *A. conyzoides*, and it is also revealed that having a higher amount of chlorophyll-*a* and chlorophyll-*b* is an indication of higher total chlorophyll content.

### 2.4. Effect of P. hysterophorus Extract on Carotenoid Content of the Test Plant Species

Carotenoid contents of *A. conyzoides*, weedy rice and *C. iria* were significantly affected by the foliar application of *P. hysterophorus* extract at different exposure times (Table 2). At 6 HAS *P. hysterophorus* extract reduced the carotenoid contents of *A. conyzoides*, weedy rice and *C. iria* from 16.82 to 32.89%, 2.65 to 10.82% and 3.84 to 11.41%, respectively at lowest (20 g L^−1^) to highest (60 g L^−1^) concentration compared to the control (0 g L^−1^). The highest inhibition was started to occur for all test plants at 24 HAS, while the lowest carotenoid content was observed in *A. conyzoides* compared to weedy rice and *C. iria*. Carotenoid contents showed a decline of 71.29%, 32.45% and 35.32% in *A. conyzoides*, weedy rice and *C. iria*, respectively, at 72 HAS in the presence of the highest concentration of *P. hysterophorus.*

### 2.5. Effect of P. hysterophorus Extract on Photosynthesis Rate of the Test Plant Species

The significant interaction effect of time after spray and extract concentration was observed on the photosynthesis rate of *A. conyzoides*, weedy rice and *C. iria* (Table 3). The different exposure times of *P. hysterophorus* extract significantly decreased the rate of photosynthesis of test plants. The extract levels had triggered different responses by the species, and this could be attributed to the inherent genetic variability among the species. Nevertheless, *A. conyzoides* had expressed more reduction in photosynthetic rate across the species with inhibition of 21.73%, while 7.82% and 10.54% were observed in weedy rice and *C. iria*, respectively at 6 HAS with the highest (60 g L^−1^) concentration of *P. hysterophorus*. Among the times of spray, photosynthesis rate was more reduced at 72 HAS in *A. conyzoides* at higher (60 g L^−1^) concentration of extract with an inhibition index of 78.85%, followed by 36.33% and 28.37% in *C. iria* and weedy rice, respectively.

### 2.6. Effect of P. hysterophorus Extract on Stomatal Conductance of the Test Plant Species

A significant effect was observed on stomatal conductance of *A. conyzoides*, weedy rice among different hours after spray (Table 3). The values of different exposure times in *C. iria* were not significantly different. There is a discernible difference in response to the extract concentration levels at a time after spray among the test species. Lower stomatal conductance was recorded in *A. conyzoides* (0.35 mol m^−2^ s^−1^), then *C. iria* (0.38 mol m^−2^ s^−1^) and weedy rice (0.53 mol m^−2^ s^−1^), and this indicated a remarkable variation in the integral phenomena of the test plant species. However, a minimum inhibition index of only 6.34% and 6.96% appeared in *C. iria* and weedy rice, respectively, while *A. conyzoides* was possessed the highest (20.38%) inhibition at 6 HAS at the highest (60 g L^−1^) concentration. The highest inhibition was started at the highest (60 g L^−1^) concentration of *P. hysterophorus* extract at 24 HAS with an inhibition index of 59.54%, 22.20% and 25.45% in *A. conyzoides* weedy rice and *C. iria*, respectively.

### 2.7. Effect of P. hysterophorus Extract on Transpiration Rate of the Test Plant Species

Foliar spray of *P. hysterophorus* extract had a significant effect on the transpiration rate of *A. conyzoides*, weedy rice and *C. iria* (Table 3). The *P. hysterophorus* extract at 20, 40, and 60 g L^−1^ concentrations resulted in 5.84%, 9.27% and 20.66% reduction at 6 HAS, while 25.28%, 55.09% and 78.94% reduction at 72 HAS in transpiration rate than control (0 g L^−1^), respectively for *A. conyzoides*. Weedy rice and *C. iria* were less sensitive to the extract concentration of *P. hysterophorus* compared to *A. conyzoides*. At 24 HAS, transpiration rate showed a reduction of 78.81%, 34.69% and 28.53% in *A. conyzoides*, *C. iria* and weedy rice, respectively, by the foliar application of the maximum (60 g L^−1^) concentration of *P. hysterophorus*. The lowest transpiration rate was observed for weedy rice (9.67 mmol H_2_O m^−2^ s^−1^) and *C. iria* (6.95 mmol m^−2^ s^−1^) at 72 HAS at the highest (60 g L^−1^) concentration, and the inhibition was 31.32% and 38.48%, respectively.

### 2.8. Effect of P. hysterophorus Extract on Malondialdehyde Content of the Test Plant Species

**Figure 1:**
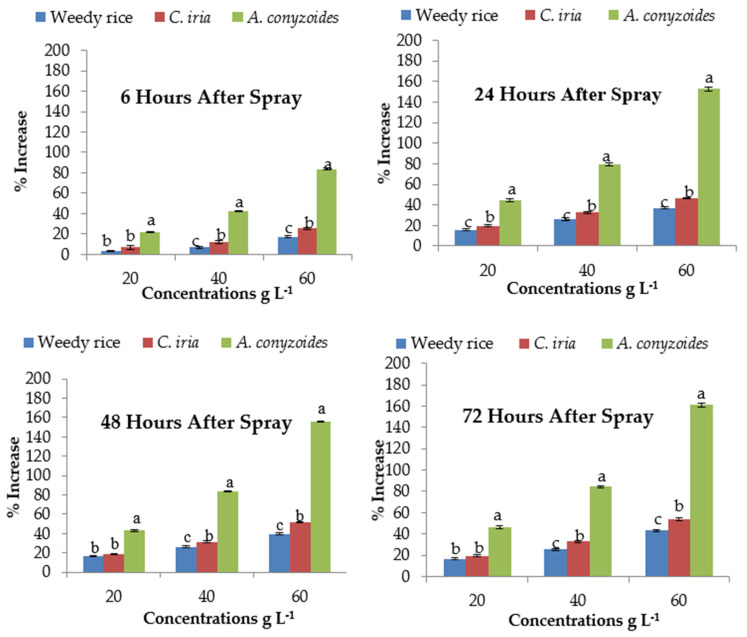
Malondialdehyde (MDA) content (% increase compared with control) of test weeds treated with *P. hyster0ophorus* extract concentrations at the different exposure times. Values with the same letter among the test weeds at the same extract concentrations are not significantly different at *p* > 0.05 by Tukey’s HSD.

Malondialdehyde (MDA) content was significantly lower in control for all the test plant species compared to the treated plants with the *P. hysterophorus* extract (Table 4). MDA content showed a concentration-dependent increase in response to *P. hysterophorus* extract. At 6 HAS, MDA content of *A. conyzoides* was increased by 21.99 to 83.70% at the lowest (20 g L^−1^) and highest (60 g L^−1^) concentrations, respectively compared to control (0 g L^−1^), and that for weedy rice and *C. iria* the values were 3.72 to 17.15% and 6.74 to 25.42%, respectively (Figure 1). The MDA level of test weeds was unchanged after short exposure (i.e., 6 HAS) at the lowest concentration of *P. hysterophorus*. The MDA contents were significantly higher at long exposure time (24, 48 and 72 HAS) than short exposure time (6 HAS) to *P. hysterophorus* extract (Figure 1). The MDA levels of *A. conyzoides* were 155.66% higher than the control (0 g L^−1^) at 48 HAS with the highest (60 g L^−1^) concentration of *P. hysterophorus* extract.

### 2.9. Effect of P. hysterophorus Extract on Proline Content of the Test Plant Species

**Figure 2:**
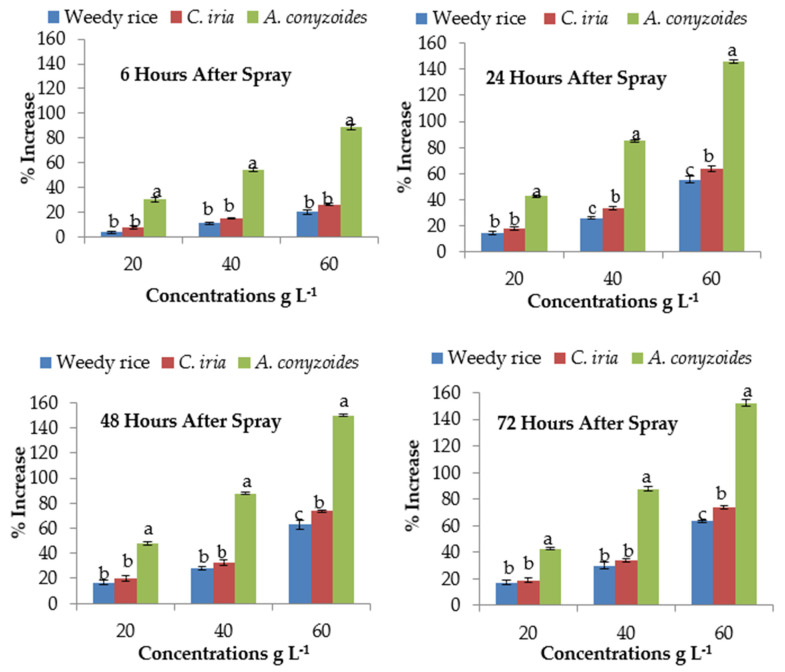
Proline content (% increase compared with control) of test weeds treated with *P. hysterophorus* extract concentrations at the different exposure times. Values with the same letter among the test weeds at the same extract concentrations are not significantly different at *p* > 0.05 by Tukey’s HSD.

The proline content was calculated at different exposure times (6, 24, 48 and 72 HAS) and exhibited a significant difference (Table 4). After 6 HAS, the proline content of *A. conyzoides* was increased by 30.32% compared to control (0 g L^−1^) at the lowest (20 g L^−1^) concentration, while at the same time, only 4.15% and 7.92% increase occurred in weedy rice and *C. iria*, respectively (Figure 2). The highest (60 g L^−1^) concentration of *P. hysterophorus* extract exhibited 146.28%, 149.73% and 150.31% higher proline content than control (0 g L^−1^) at 24, 48 and 72 HAS in *A. conyzoides*, respectively. The increased percentages of proline contents of weedy rice (55.27%) and *C. iria* (63.66%) were lower than that of *A. conyzoides* (130.32%) at 24 HAS with the highest (60 g L^−1^) concentration compared to control (0 g L^−1^).

### 2.10. Effect of P. hysterophorus Extract on Superoxide Dismutase of the Test Plant Species

**Figure 3:**
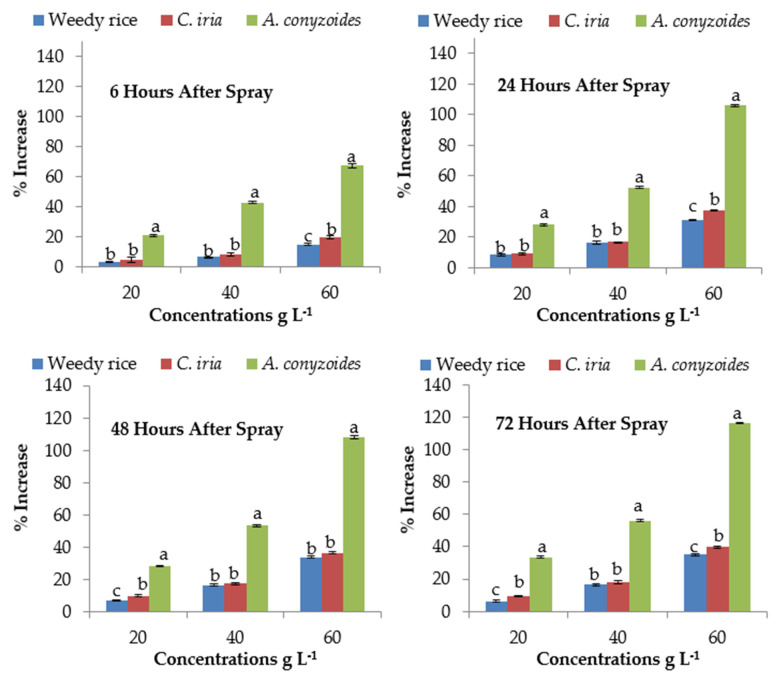
Activities of superoxide dismutase (SOD) (% increase compared with control) of test weeds treated with *P. hysterophorus* extract concentrations at the different exposure times. Values with the same letter among the test weeds at the same extract concentrations are not significantly different at *p* > 0.05 by Tukey’s HSD.

Foliar spray of *P. hysterophorus* extract significantly affected the activity of superoxide dismutase (SOD) enzyme of *A. conyzoides*, weedy rice and *C. iria* (Table 5). The SOD activity was found the highest with the application of the highest (60 g L^−1^) amount of *P. hysterophorus* extract. In *A. conyzoides*, the values were 67.29%, 105.87%, 108.31% and 116.26%, in weedy ricevalueswere14.78%, 31.21%, 34.07% and 35.06%, and in *C. iria* they were 19.61%, 37.48%, 36.72% and 39.70% at 6, 24, 48, 72 HAS, respectively (Figure 3). However, the SOD content of *A. conyzoides* was about 68 to 71% higher than weedy rice and *C. iria*, respectively, at 24 HAS at the highest (60 g L^−1^) concentration. At the highest concentration (60 g L^−1^), the maximum SOD activity of *A. conyzoides* was 1.67 to 2.16 times higher than the control (0 g L^−1^) of different exposure times (6, 24, 48 and 72). At 72 HAS, the SOD activity of weedy rice and *C. iria* was increased by 6.44 to 35.06% and 9.38 to 39.70% at the lowest (20 g L^−1^) and highest (60 g L^−1^) concentration compared to control (0 g L^−1^), respectively.

### 2.11. Effect of P. hysterophorus Extract on Catalase Activity of the Test Plant Species

**Figure 4:**
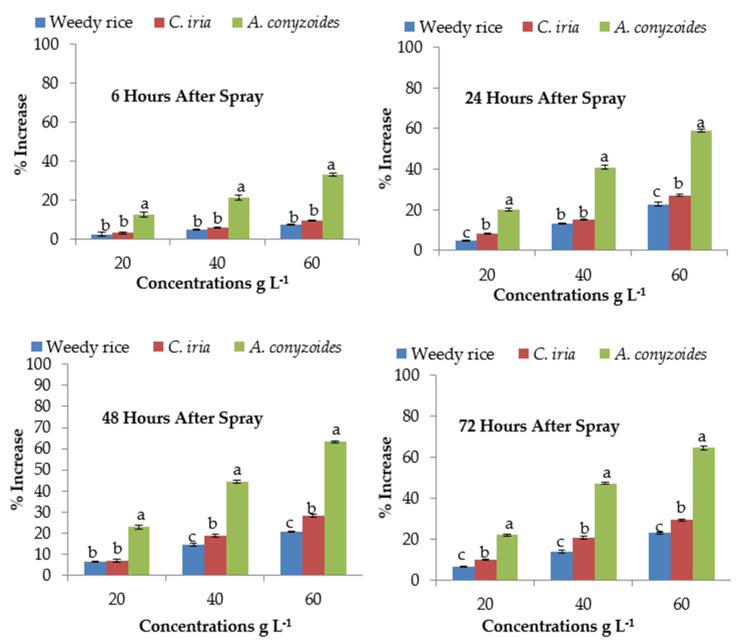
Activities of catalase (CAT) (% increase compared with control) of test weeds treated with *P. hysterophorus* extract concentrations at the different exposure times. Values with the same letter among the tested weed at the same extract concentrations are not significantly different at *p* > 0.05 by Tukey’s HSD.

Catalase (CAT) activity differed significantly with increasing *P. hysterophorus* extract concentration at different exposure times except for weedy rice (Table 5). At the lowest (20 g L^−1^) concentration, CAT activity of *A. conyzoides* showed 12.47, 20.04, 22.99 and 22.08% increases after 6, 24, 48 and 72 HAS respectively compared to control (0 g L^−1^). These activities increased to 1.33, 1.59, 1.63 and 1.64 times than that of the control (0 g L^−1^) at the highest (60 g L^−1^) *P. hysterophorus* concentration after 6, 24, 48 and 72 h, respectively in *A. conyzoides*. The CAT activity of weedy rice was increased only 2.46 to 7.40% at 6 HAS, while 6.80% and 23.06% increases occurred at 72 HAS in the lowest (20 g L^−1^) and highest (60 g L^−1^) concentration, respectively, compared to control (0 g L^−1^) (Figure 4). The highest CAT activity was observed at 72 HAS for *C. iria* with the highest amount of *P. hysterophorus*, and the increasing percentage was 29.39. No significant differences were recorded in *C. iria* and weedy rice among different exposure times of *P. hysterophorus* extract concentrations.

### 2.12. Effect of P. hysterophorus Extract on Peroxidase Activity of the Test Plant Species

**Figure 5:**
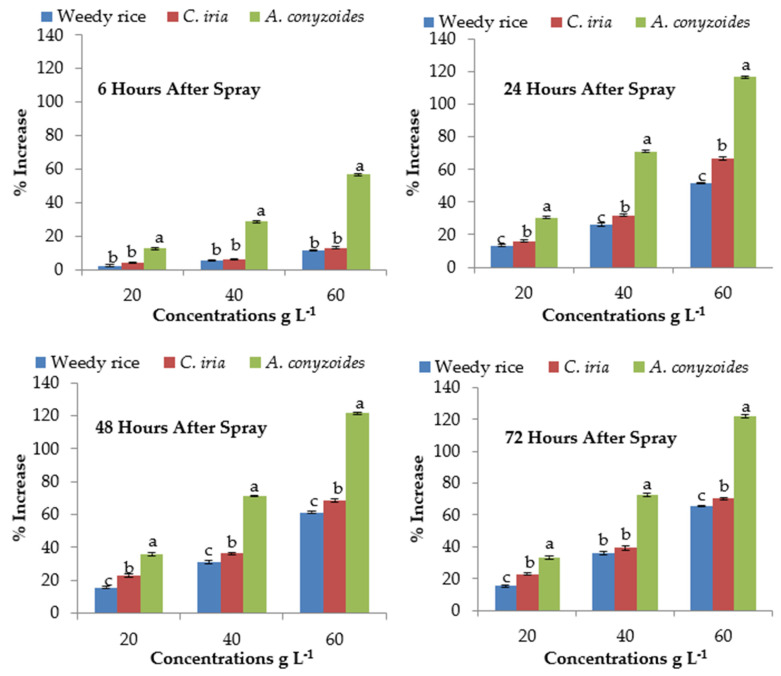
Activities of peroxidase (POD) (% increase compared with control) of test weeds treated with *P. hysterophorus* extract concentrations at the different exposure times. Values with the same letter among the tested weed at the same extract concentrations are not significantly different at *p* > 0.05 by Tukey’s HSD.

The interaction of exposure time and *P. hysterophorus* extract significantly influenced the peroxidase (POD) activity of *A. conyzoides*, weedy rice and *C. iria* (Table 5). The POD activity of all test plant species was significantly different at different exposure times. Longer exposure times of spray (i.e., 24, 48 and 72 h) induced a significant increase in the POD activity (Figure 5). The POD activity increased significantly with the increase in concentration where the greatest activity (116.43%) was recorded in 60 g L^−1^, while 40 and 20 g L^−1^ concentrations recorded 70.91% and 30.50% increase at 24 HAS in *A. conyzoides* compared to control. On the other hand, at 24 HAS, POD activities of weedy rice and *C. iria* were increased by 51.63% and 66.71%, respectively, at the highest (60 g L^−1^) concentration of *P. hysterophorus* extract compared to control (0 g L^−1^). The increased percentage of POD activity was 33.30 to 121.94%, 15.39 to 65.59% and 22.88 to 70.39% in *A. conyzoides*, weedy rice and *C. iria*, respectively, at the highest exposure time (72 HAS) compared to control.

## 3. Discussion

*Parthenium hysterophorus* has been spreading like wildfire in different countries and creates harm, including threats to biodiversity, allergies, mutagenesis and dermatitis in livestock and humans. In this study, we observed the effect of *P. hysterophorus* extract on physiological and biochemical changes in *A. conyzoides*, weedy rice and *C. iria* at different times of exposure.

Changes in chlorophyll levels in leaves are an important indicator of plant resistance and environmental quality [20]. In this study, we found a significant decrease in the treatment groups compared to the control groups. This decrease in the chlorophyll content is due to the allelopathic effect of *P. hysterophorus*. The decreased chlorophyll and carotenoid contents depend on the concentration of *P. hysterophorus* extract, and these tallies with the finding of Ding et al. [21], who observed that a high concentration of allelochemicals inhibits photosynthesis and plant growth by reducing chlorophyll content. Yilmaz et al. [22] and Erdal [23] have explained that decreased chlorophyll content during stress conditions is associated with an increase in the chlorophyllase enzyme. Carotenoids served as an antioxidant against free radicals and photochemical damage [24]. Chlorophyll *b* and carotenoids were decreased by 81.40% and 77.8% when exposed to *Portulaca oleracea* root extract, and these could be attributed to a decrease in chlorophyll biosynthesis or degradation of existing chlorophyll [25].

Foliar spray of *P. hysterophorus* extract reduced photosynthesis rate, stomatal conductance and transpiration of *A. conyzoides*, weedy rice and *C. iria*. Reduction in leaf photosynthesis was attributed to a decrease in photosynthetic metabolites, carboxylation efficiency, impairment of chloroplast activity, increase in enzyme activities [26], and production of ROS caused impediment of photosynthetic mechanism [27]. Stomatal control is an important property through which the plants limit the loss of water, affecting gas changes. This characteristic can be influenced by several factors, including stress [28], and it can be an indication of lower photosynthetic efficiency. The efficiency of using water and carboxylation was also reduced in the plants subjected to the application of *P. hysterophorus* extract.

The reduction in the transpiration rate is certainly associated with stomatal conductance. This study reveals that *P. hysterophorus* extract played a notable role in decreasing the transpiration rate for test plants at different exposure times. The concentration of phenolic acids resulted in a decline in overall water utilization and transpiration of cucumber seedlings in a linear manner [29]. The solution of cinnamic acid and benzoic acids decreased stomatal conductance and transpiration of cucumber seedlings [30]. The results obtained for the variables related to photosynthesis corroborate the ones obtained for phytotoxicity, as phytotoxicity caused by the increased amount of *P. hysterophorus* extract and exposure time, there was a reduction in the photosynthesis, stomatal conductance and transpiration rate.

In the present study, *P. hysterophorus* exposures increase the activities of SOD, POD and CAT. SOD is the first step in the removal of ROS. Specifically, it converts O_2_^−^ to H_2_O and oxygen. Therefore, the increases in the activity of SOD in response to *P. hysterophorus* suggest an increased production of O_2_^−^. Similarly, the increased activities of POD and CAT indicate potential protection against oxidation by this antioxidant enzyme [31]. In this study, SOD, POD and CAT activities in the leaves of *A. conyzoides* were significantly increased than weedy rice and *C. iria* at higher concentrations. The activities of one or more of these enzymes generally increase when plants are exposed to stressful conditions [32]. Ding et al. [21] reported the toxicity of allelochemicals from garlic root exudates on the activity of protective enzymes might be due to their intra-structure disruption.

In this study, the enzymatic activities of *A. conyzoides* at 72 HAS were stimulated significantly at higher concentrations to 64.41% and 120.18%, respectively. The high overwhelming increase in POD indicated an abundant high level of H_2_O_2_, which is a byproduct of SOD metabolism. Generally, the activity of the enzyme increases with increasing *P. hysterophorus* extract concentration. It appears that the enzymes (SOD, CAT and POD) were not adequate to scavenge ROS and prevent membrane oxidation in the organelles, especially mitochondria and chloroplast, at the initial period of *P. hysterophorus* extract application. In addition, chlorotic symptoms revealed signal molecules activation and secretion of defense molecules against allelochemical stress. The recovery action observed in *A. conyzoides* signifies the rapid production of ROS.

An increased malondialdehyde (MDA) level in the plant tissue represents an important indicator of membrane lipid peroxidation. In our study, the MDA content increase in *P. hysterophorus* sprayed plants compared to control (without extract) plants, and this increase was more apparent in the highest concentration. *P. hysterophorus* may induce the formation of free radicals by inducing the degradation of carotenoids and the indirect degradation of chlorophyll. Furthermore, increased free radicals level can increase membrane lipid peroxidation and thus MDA content. Saidi et al. [33] reported that the bean plants exposed to 20 μM cadmium increased MDA levels compared to control plants. Choudhury and Panda [34] stated that an increase in MDA content is an indicator of oxidative stress. These findings are consistent with our result. The consistently high level of MDA suggests that the antioxidant enzymes induced by glufosinate may not be able to completely eliminate ROS within a short period of time.

Proline is the amino acid that is associated with different stresses in the plant. Under abiotic stress, accumulation of proline in the plant may be an adaptive and metabolic measure of stress, an inhibitor of lipid peroxidation and defense against toxicity [35]. Plants develop mechanisms for the accumulation of compatible solutes, such as betaine, sugar, polyol and proline to counteract various abiotic stress, and among them, proline is the most important solutes that curtail the impact of osmotic adjustment [36]. The greatest content of proline in 72 HAS was observed in the treatment with *P. hysterophorus*. Weedy rice and *C. iria* had the lowest proline content compared to *A. conyzoides*. This was expected considering stress and leaf damage that was visually observed during experimentation. In a similar relation, foliar application of *Medicago sativa* leaf extracts significantly raised the proline content of three wheat varieties [37]. Proline content of tomato, wheat and cucumber treated with aqueous extract of *Calotropis procera* were stimulated with an increase in concentration [38].

## 4. Materials and Methods

### 4.1. Experimental Site

The experiment was conducted in a glasshouse in Farm 15 at the Faculty of Agriculture Universiti Putra Malaysia (3°02′ N, 101°42′ E, 31 m elevation). The climate was hot (on average 36−38 °C) and humid with abundant rainfall during the experimental period.

### 4.2. Test Plants

Three weed species, namely *A. conyzoides* (*voucher specimen#UPMWS001*), *C. iria* (*voucher specimen#UPMWS019*) and weedy rice (*voucher specimen#UPMWS025*), were used as test plants. The seeds of weedy rice were collected from the rice field of Sekinchan, Kuala Selangor, Selangor, Malaysia, and other weed seeds (*A. conzyoides* and *C. iria*) were collected from farm 15, Universiti Putra Malaysia. The voucher specimens are deposited in the Weed Science Laboratory, Department of Crop Science, Faculty of Agriculture, Universiti Putra Malaysia.

### 4.3. Preparation of P. hysterophorus Extract for Foliar Spray

The whole plant of *P. hysterophorus* at the maximum vegetative stage was collected. The collected weed was washed carefully with running tap water to remove the dust particles, then air-dried in open trays under shade at room temperature for 3 weeks. Then chopped and powdered in a Willey mill. In a conical flask, 100 g powder of *P. hysterophorus* was soaked with 1000 mL methanol: distilled water (80:20, *v*/*v*) and paraffin were used for wrapping the flask. The flask was shaken in an orbital shaker at 150 rpm agitation speed for 48 h at room temperature (24−26 °C). The solution was filtered using four layers of cheesecloth, then centrifuged for 1 h at 3000 rpm, then re-filtered using 0.2 μm, 15 mm syringe filters (Phenex, Nonsterile, Luer/Slip, LT Resources, Malaysia). The collected supernatant was evaporated by a rotary evaporator at 40 °C. The dried residue was weighed and converted to % as under:

Extraction percentage = [Extract weight (g)/powder weight (g)] × 100%
(1)

Each stock extract was diluted with sterile distilled water to get 20, 40, 60 g L^−1^ concentrations of extract for bioassay. All extracts were stored in the dark at 4 °C in the refrigerator until used. The methanol extracts were prepared as per the method of Motmainna et al. [39].

### 4.4. Experimental Layout

Pre-germinated seeds of *A. conyzoides*, weedy rice and *C. iria* were seeded in each pot (15 cm diameter) and then covered with soil at a depth of 1 cm, and finally, the soil was moistened with tap water. After germination, equal-sized healthy five seedlings were maintained in each pot. The pots were arranged in a randomized complete block design with four replications. The methanol extract of *P. hysterophorus* was sprayed with 20, 40 or 60 g L^−1^ concentration on tested plants (4–6 for broadleaf and 2–3 leaf stage for grasses and sedges species) with the help of a hand-operated atomizer at the rate of 100 mL m^−2^ [40]. Plants in the control treatment were sprayed with 200 mL water without extract at two-day intervals or when needed.

### 4.5. Data Collections

The physiological and biochemical changes due to the application of *P. hysterophorus* extract on the test weed species were observed to elucidate the possible mechanism of its allelopathy data on photosynthesis rate, transpiration and stomatal conductance, chlorophyll fluorescence, chlorophyll pigments, proline and antioxidant enzymes. Mature leaves of each test weed species were plucked at 6, 24, 48 and 72 h after spray and kept inside aluminum foil and brought to the laboratory from glasshouse through icebox. The leaf samples were harvested between 8 to 10 am and rapidly frozen with liquid nitrogen and then stored at −80 °C for further biochemical analysis.

#### 4.5.1. Photosynthesis Rate, Transpiration and Stomatal Conductance

The rate of photosynthesis, transpiration and stomatal conductance were measured from randomly selected four leaves from each test weed species using LICOR (LI-6400XT) portable photosynthesis system (LI-COR-Inc Lincoln, Lincoln, NE, USA) between 9.00 am to 11.00 am under bright daylight. The measurements were taken on the abaxial surface at a CO_2_ flow rate of 400 μmol m^−2^ s^−1,^ and the saturating photosynthetic photon flux density (PPFD) was 1000 mmol m^−2^ s^−1^ [36].

#### 4.5.2. Chlorophyll Pigments

The total chlorophyll and carotenoids contents were determined following the procedure of Lichenthaler and Bushman [41] and Amin [42]. The samples of the fresh leaf (0.1 g) were homogenized into a glass bottle having an amount of 10 mL of 80% acetone. The glass bottles were covered by aluminum foil and kept in the dark place for three to four days at room temperature. After finishing the incubation, the test tubes were vortexed and needed to wait until the sediments have found at the bottom. The spectrophotometric reading (UV-3101 P, Labomed Inc., Angeles, CA, USA) of the absorbance of the solution was measured at 663.2 nm, 646.8 nm and 470 nm, and as a blank, 80% acetone was used. Chlorophyll-*a*, chlorophyll-*b*, total chlorophyll and carotenoids contents were expressed as mg g^−1^ of fresh weight (FW) using the following relationships:

Chlorophyll-*a* (μg mL^−1^) = (12.25 × A663.2 − 2.79 × A646.8) (2)

Chlorophyll-*b* (μg mL^−1^) = (21.50 × A646.8 − 5.1 × A663.2) (3)

Total chlorophyll (μg mL^−1^) = (7.15 × A663.2 + 18.71 × A646.8) (4)

(5) $$\mathrm{Carotenoids}\;(\mu g\;{\mathrm{mL}}^{-1})=\frac{\left(1000\times A470-1.8\times \mathrm{chl}a-85.02\times \mathrm{chl}b\right)}{198}$$

#### 4.5.3. Malondialdehyde (MDA) Content

The malondialdehyde (MDA) content was determined by the following protocol as previously described by Robert [43]. An amount of 0.2 g of grounded leaf tissue was homogenized in 2 mL ultrapure distilled water. Then the samples were centrifuged (Sigma 3K30) at 10,000 rpm for 15 min. One milliliter of this solution and 2 mL of thiobarbituric acid (TBA)/trichloroacetic acid (TCA) (Merck, German) solution (0.5% TBA in 20% TCA) were boiled in a water bath at 90 ℃ for 30 min. After boiling, the test tubes containing the solution were cooled in an ice bath. The final mixture was centrifuged again at 10,000 rpm for 15 min. The spectrophotometric absorbance (UV-3101PC, Shimadzu) of the supernatants was recorded at 450, 532 and 600 nm. The MDA concentration was measured using the following relationship:

MDA (μM) = {6.45 × (D532 − D600) − 0.56 × D450}(6)

where D450, D532 and D600 were the absorbances at 450, 532 and 600 nm, respectively. The MDA content was finally expressed as µmol g^−1^ FW.

#### 4.5.4. Proline Content

Proline measurement protocol was done according to methods described by Bates et al. [44] with slight modifications. An amount of 0.1 g of fresh leaves was homogenized with the presence of 2 mL of 5% (*w*/*v*) sulfosalicylic acid. Sample containing test tubes were centrifuged at 10,000 rpm for 10 min. One milliliter of the supernatant was added in one milliliter of acid ninhydrin (1.25 g of ninhydrin; 30 mL of glacial acetic acid; 20 mL of phosphoric acid 6 M) and one milliliter of glacial acetic acid. Then the solution containing tubes was incubated at 95 °C for one hour in a water bath and cooled for 10 min in an ice bath. Two milliliters of toluene was added in each tube with samples then shaken using a vortex. The proline concentration was recorded by taking the absorbance reading at 520 nm using the microplate reader (Bio Tek 800 TS). The content of proline was calculated by plotting the value in a standard curve considering L-proline as standard (Sigma-Aldrich, St. Louis, MO, USA), then proline content was calculated following the equation stated below:

(7) $$\mathrm{Proline}\;\left({\mu \mathrm{molg}}^{-1}\;\mathrm{FW}\right)=\frac{\frac{\mathrm{Proline}\;\left({\mu g\;\mathrm{mL}}^{-1}\right)\times \mathrm{Tolune}\;\left(\mathrm{mL}\right)}{115.5\;\left({\mu g\;\mu \mathrm{mole}}^{-1}\right)}}{\mathrm{Freshweightofsample}\;\left(g\right)}$$

where the molecular weight of proline is 115.5 (μg μmole^−1^).

#### 4.5.5. Enzymes Extraction

The enzyme extraction was performed based on the protocol described by Gupta et al. [45] to determine the activity of the antioxidant enzymes, superoxide dismutase (SOD), catalase (CAT), and peroxidase (POD). Fresh leaf sample was ground in a porcelain mortar with liquid nitrogen. Then 0.1 g ground leaf sample was taken into 2 mL Eppendorf tube, and 1.5 mL potassium phosphate buffer was added. The mixture was centrifuged at 10,000 rpm for 20 min.

##### Determination of SOD

The reaction mixture was prepared by adopting the method described by Gupta et al. [45]. The reaction mixture contained 0.05 mL of the enzyme extract, 1.5 mL of 100 mM potassium phosphate buffer, 0.1 mL of 3 mM EDTA, 0.1 mL of 200 mM methionine, 0.01 mL 2.25 mM NBT (n-nitro blue tetrazolium) and 1 mL of ultra-pure distilled water. Finally, 60 μM of riboflavin was added into each reaction mixture in the dark. Then the tubes were incubated in a 15 watts fluorescent lamp for 10 min. The control mixture without enzyme extract was kept under the light. The blank consisted of reaction mixtures with no enzyme extract and without keeping under the light to stop the reaction immediately after incubation. Aluminum foil was used to cover the tubes; SOD was measured using a spectrophotometer (UV-3101PC) at 560 nm through recording the change in absorbance due to the reaction of the superoxide nitro blue tetrazolium complex with enzyme extracts. Each unit of enzyme activity was calculated by the amount of enzyme that inhibits NBT reduction to 50% using the formula described as follows:

The activity of SOD can be expressed as unit mg^−1^ FW:

(8) $$\mathrm{SOD}\;(\%\;\mathrm{inhibition})=\frac{\left(A560\;\mathrm{control}-A560\;\mathrm{sample}\right)\times 100}{A560\;\mathrm{control}}$$

(9) $$\mathrm{SOD}\;(\mathrm{unit}\;{\mathrm{mL}}^{-1})=\frac{\%\;\mathrm{inhibition}\times \mathrm{total}\;\mathrm{volume}}{50\times \mathrm{enzyme}\;\mathrm{volume}}$$

(10) $$\mathrm{SOD}\;(\mathrm{unit}\;{\mathrm{mg}}^{-1}\;\mathrm{FW})=\frac{{\mathrm{unit}\;\mathrm{mL}}^{-1}}{\mathrm{enzyme}\;\left({\mathrm{mg}\;\mathrm{mL}}^{-1}\right)}$$

where the absorbance of control and sample was recorded by 560 nm at 1 min, 50% inhibition is equal to 1 unit of SOD production.

##### Determination of CAT

The CAT activity was studied considering the methodology stated by Aebi [46]. A 3.0 mL of reaction mixture contained 1.5 mL of 100 mM potassium phosphate buffer, 0.5 mL of 75 mM hydrogen peroxide (H_2_O_2_), 0.05 mL of enzyme extract and 0.95 mL of ultrapure distilled water. The mixture with no enzyme extract was considered blank. To reach the temperature equilibration, the blank solution was put in a spectrophotometer for 4 to 5 min. The absorbance reading in a spectrophotometer (UV-3101PC, Shimadzu, Japan) at a wavelength of 240 nm was done for 2 min. Each unit of catalase enzyme activity was considered as the amount that decomposes 1 μM H_2_O_2_. The unit of catalase action was expressed as per g of fresh tissue (μMmin^−1^ g^−1^ FW).

(11) $$\mathrm{CAT}\;(\mu \mathrm{mol}\;\min^{-1}\;{\mathrm{mL}}^{-1})=\frac{\left(A240/\min\right)\times \mathrm{total}\;\mathrm{volume}\;\times 1000}{43.6\times \mathrm{enzyme}\;\mathrm{volume}}$$

(12) $$\mathrm{CAT}\;(\mu \mathrm{mol}\;\min^{-1}\;{\mathrm{mg}}^{-1}\;\mathrm{FW})=\frac{\mu \mathrm{mol}\;\min^{-1}{\mathrm{mL}}^{-1}}{\mathrm{enzyme}\;\left({\mathrm{mg}\;\mathrm{mL}}^{-1}\right)}$$

where the absorbance of the sample was recorded by 240 nm at 1 min, here the extinction coefficient is 43.6.

##### Determination of POD

The POD activity was determined according to the methodology described by Rao et al. [47] and measured by the oxidation of guaiacol at 470 nm by H_2_O_2_. A reaction mixture of 3 mL consisted of 0.1 mL of enzyme extract, 0.05 mL of 20 mM guaiacol and 2.83 mL of 10 mM phosphate buffer (pH 7.0). For starting the reaction, 0.02 mL of 40 mM H_2_O_2_ was added. The mixture with no enzyme extract was considered blank. To reach the temperature equilibration, the blank solution was put in a spectrophotometer for 4 to 5 min. The absorbance reading was taken in the wavelength at 470 nm, and the unit of POD was expressed in μmol min^−1^ g^−1^ FW.

(13) $$\mathrm{POD}\;(\mu \mathrm{mol}\;\min^{-1}\;{\mathrm{mL}}^{-1})=\frac{\left(A470/\min\right)\times \mathrm{total}\;\mathrm{volume}\;\times 1000}{26.6\times \mathrm{enzyme}\;\mathrm{volume}}$$

(14) $$\mathrm{POD}\;(\mu \mathrm{mol}\;\min^{-1}\;{\mathrm{mg}}^{-1}\;\mathrm{FW})=\frac{\mu \mathrm{mol}\;\min^{-1}{\mathrm{mL}}^{-1}}{\mathrm{enzyme}\;\left({\mathrm{mg}\;\mathrm{mL}}^{-1}\right)}$$

where the absorbance of the sample was recorded by 470 nm at 1 min, here the extinction coefficient is 26.6.

### 4.6. Statistical Analysis

A randomized complete block design (RCBD) with four replications was adopted. Data were analyzed using SAS 9.4 software. The mean comparison was conducted using Tukey’s test at a 5% probability level.

## 5. Conclusions

The result from this study suggests that allelochemicals present in the methanol extract of *P. hysterophorus* were responsible for the changes of physiological and biochemical parameters of *A. conyzoides*, weedy rice and *C. iria*. The extract concentrations caused the reduction in chlorophyll content, carotenoids and subsequently hindered the photosynthesis rate. The activity of antioxidant enzymes (SOD, CAT and POD), MDA and proline content was induced and stimulated by foliar spray of *P. hysterophorus* in a concentration-dependent pattern. *Ageratum conyzoides* maintained comparatively low stress tolerance compared to weedy rice and *C. iria*. This situation offers the opportunity to control weeds that are resistant to the present herbicides. With the new tools of molecular genetics, proteomics, metabolomics profiling, modern and sophisticated methods of chemistry and biochemistry, we may develop new compounds based on the structure of potential natural herbicidal compounds from *P. hysterophorus* to formulate selective and eco-friendly herbicides.

## Figures and Tables

**Table 1:** Response of chlorophyll-*a* content and chlorophyll-*b* of *A. conyzoides*, weedy rice and *C. iria* to a foliar spray of *P. hysterophorus* extract.

Test Plants	Dose (g L^−1^)	Chlorophyll-*a* (mg g^−1^ FW)				Chlorophyll-*b* (mg g^−1^ FW)			
		Hours after Spray				Hours after Spray			
		6	24	48	72	6	24	48	72
	0	3.03a ± 0.04 (0)	3.05a ± 0.06 (0)	2.91a ± 0.11 (0)	3.06a ± 0.07 (0)	1.14a ± 0.08 (0)	1.16a ± 0.09 (0)	1.11a ± 0.12 (0)	1.31a ± 0.13 (0)
	20	2.65a ± 0.11 (12.50)	2.13b ± 0.07 (30.01)	2.04b ± 0.04 (29.78)	2.11b ± 0.10 (31.10)	1.01a ± 0.03 (13.53)	0.86a ± 0.22 (25.55)	0.82a ± 0.07 (26.54)	0.96a ± 0.07 (26.45)
*A. conyzoides*	40	2.42a ± 0.03 (20.20)	1.63b ± 0.04 (46.49)	1.57b ± 0.03 (45.90)	1.64b ± 0.10 (46.54)	0.95a ± 0.07 (18.29)	0.67b ± 0.14 (42.20)	0.63b ± 0.07 (43.27)	0.74b ± 0.06 (43.27)
	60	2.06a ± 0.07 (31.98)	0.78b ± 0.10 (74.32)	0.77b ± 0.03 (73.53)	0.79b ± 0.04 (74.09)	0.82a ± 0.13 (29.56)	0.38b ± 0.03 (67.50)	0.34b ± 0.05 (68.95)	0.39b ± 0.06 (70.44)
	0	3.89a ± 0.08 (0)	3.86a ± 0.10 (0)	3.90a ± 0.16 (0)	3.95a ± 0.12 (0)	2.37a ± 0.17 (0)	2.23a ± 0.17 (0)	2.12a ± 0.22 (0)	2.15a ± 0.20 (0)
	20	3.80a ± 0.08 (2.33)	3.45b ± 0.07 (10.61)	3.37c ± 0.10 (13.59)	3.18c ± 0.03 (19.34)	2.32a ± 0.02 (2.01)	2.01b ± 0.08 (9.74)	1.94b ± 0.20 (8.30)	1.92b ± 0.13 (10.75)
Weedy rice	40	3.59a ± 0.04 (7.70)	3.06b ± 0.10 (20.86)	3.11b ± 0.09 (20.37)	3.10b ± 0.10 (21.41)	2.20a ± 0.16 (6.97)	1.85b ± 0.09 (16.87)	1.75b ± 0.11 (17.51)	1.75b ± 0.11 (17.93)
	60	3.39a ± 0.03 (12.80)	2.56b ± 0.06 (33.75)	2.59b ± 0.05 (33.73)	2.52b ± 0.05 (36.21)	2.11a ± 0.09 (10.96)	1.51b ± 0.06 (32.08)	1.43b ± 0.09 (32.23)	1.47b ± 0.07 (31.68)
	0	3.90ab ± 0.07 (0)	3.78b ± 0.05 (0)	3.86ab ± 0.09 (0)	3.99a ± 0.05 (0)	3.39a ± 0.09 (0)	3.07bc ± 0.12 (0)	3.29ab ± 0.10 (0)	2.83c ± 0.13 (0)
	20	3.75a ± 0.04 (3.63)	3.05b ± 0.11 (19.30)	3.10b ± 0.07 (19.65)	3.19b ± 0.07 (20.12)	3.28a ± 0.06 (3.36)	2.53b ± 0.29 (17.60)	2.68b ± 0.28 (18.53)	2.30b ± 0.06 (18.71)
*C. iria*	40	3.54a ± 0.06 (9.02)	2.79b ± 0.07 (26.09)	2.85b ± 0.07 (26.05)	2.84b ± 0.05 (28.76)	3.13a ± 0.06 (7.68)	2.39b ± 0.07 (22.24)	2.51bc ± 0.16 (23.75)	2.15c ± 0.10 (22.61)
	60	3.27a ± 0.05 (16.15)	2.42c ± 0.03 (36.03)	2.45bc ± 0.06 (36.35)	2.54b ± 0.06 (36.31)	2.99a ± 0.01 (11.72)	2.00c ± 0.07 (34.88)	2.24b ± 0.06 (31.86)	1.93c ± 0.05 (32.00)

Data are expressed as means ± SD. Values with the same letters in the row among the hours after spray for each test weed are not significantly different at *p* > 0.05. Values inside the parenthesis are inhibition percentages relative to the control.

**Table 2:** Response of total chlorophyll and carotenoids content of *A. conyzoides*, weedy rice and *C. iria* to a foliar spray of *P. hysterophorus* extract.

Test Plants	Dose (g L^−1^)	Total Chlorophyll (mg g^−1^ FW)				Carotenoids (mg g^−1^ FW)			
		Hours after Spray				Hours after Spray			
		6	24	48	72	6	24	48	72
	0	4.17ab ± 0.05 (0)	4.20ab ± 0.04 (0)	4.02b ± 0.09 (0)	4.37a ± 0.08 (0)	1.02b ± 0.03 (0)	1.03a ± 0.03 (0)	1.01a ± 0.02 (0)	1.14a ± 0.03 (0)
	20	3.66a ± 0.15 (12.79)	2.99b ± 0.18 (22.78)	2.86b ± 0.08 (28.88)	3.07b ± 0.08 (29.70)	0.86a ± 0.03 (16.82)	0.70c ± 0.02 (31.94)	0.68c ± 0.03 (32.30)	0.77b ± 0.01 (32.08)
*A. conyzoides*	40	3.37a ± 0.10 (19.67)	2.30b ± 0.11 (45.31)	2.20b ± 0.07 (45.17)	2.38b ± 0.10 (45.56)	0.81a ± 0.02 (21.01)	0.55c ± 0.01 (46.67)	0.54c ± 0.01 (46.71)	0.60b ± 0.01 (47.04)
	60	2.88a ± 0.16 (31.31)	1.16b ± 0.11 (72.45)	1.11b ± 0.07 (72.26)	1.18b ± 0.06 (73.00)	0.69a ± 0.03 (32.89)	0.30b ± 0.01 (70.94)	0.30b ± 0.01 (70.46)	0.33b ± 0.02 (71.29)
	0	6.26a ± 0.10 (0)	6.09a ± 0.10 (0)	6.02a ± 0.18 (0)	6.10a ± 0.10 (0)	1.84a ± 0.03 (0)	1.47a ± 0.70 (0)	1.78a ± 0.07 (0)	1.80a ± 0.06 (0)
	20	6.12a ± 0.07 (2.21)	5.46b ± 0.06 (10.29)	5.31bc ± 0.12 (11.73)	5.10c ± 0.13 (16.32)	1.79a ± 0.06 (2.65)	1.30c ± 0.02 (11.67)	1.53b ± 0.03 (13.64)	1.49b ± 0.07 (17.05)
Weedy rice	40	5.79a ± 0.14 (7.43)	4.91b ± 0.05 (19.40)	4.85b ± 0.15 (19.36)	4.87b ± 0.05 (20.18)	1.71a ± 0.02 (7.13)	1.15c ± 0.03 (21.89)	1.40b ± 0.09 (21.22)	1.40b ± 0.01 (22.16)
	60	5.50a ± 0.09 (12.10)	4.07b ± 0.05 (33.14)	4.02b ± 0.04 (32.20)	3.99b ± 0.10 (34.62)	1.64a ± 0.02 (10.82)	1.03c ± 0.01 (30.08)	1.24b ± 0.04 (30.01)	1.21b ± 0.02 (32.45)
	0	7.29a ± 0.06 (0)	6.85b ± 0.10 (0)	7.15a ± 0.04 (0)	6.82b ± 0.12 (0)	1.68b ± 0.03 (0)	1.73b ± 0.03 (0)	1.81a ± 0.03 (0)	1.80a ± 0.04 (0)
	20	7.03a ± 0.07 (3.51)	5.58b ± 0.24 (18.54)	5.78b ± 0.22 (19.14)	5.49b ± 0.11 (19.53)	1.61a ± 0.03 (3.84)	1.48a ± 0.06 (14.33)	1.48a ± 0.10 (18.43)	1.48a ± 0.06 (18.06)
*C. iria*	40	6.67a ± 0.1 (8.40)	5.18bc ± 0.06 (24.37)	5.36b ± 0.11 (24.99)	5.00c ± 0.14 (26.21)	1.54a ± 0.02 (8.05)	1.26b ± 0.02 (27.36)	1.32b ± 0.03 (27.43)	1.29b ± 0.15 (28.32)
	60	6.26a ± 0.03 (14.09)	4.42c ± 0.06 (35.51)	4.70b ± 0.05 (34.28)	4.47c ± 0.06 (34.52)	1.49a ± 0.01 (11.41)	1.14b ± 0.03 (34.19)	1.20b ± 0.03 (34.07)	1.17b ± 0.04 (35.32)

Data are expressed as mean ± SD. Values with the same letters in the row among the hours after spray for each test weed are not significantly different at *p* > 0.05. Values inside the parenthesis are inhibition percentages relative to the control.

**Table 3:** Response of photosynthesis rate, stomatal conductance and transpiration rate of *A. conyzoides*, weedy rice and *C. iria* to a foliar spray of *P. hysterophorus* extract.

Test Plants	Dose (g L ^−1^)	Photosynthesis Rate (µmol m^−2^ s^−1^)				Stomatal Conductance (mol m^−2^ s^−1^)				Transpiration Rate (mmol m^−2^ s^−1^)			
		Hours after Spray				Hours after Spray				Hours after Spray			
		6	24	48	72	6	24	48	72	6	24	48	72
	0	16.06a ± 0.06 (0)	14.34c ± 0.03 (0)	13.70d ± 0.01 (0)	14.60b ± 0.05 (0)	0.41a ± 0.01 (0)	0.35c ± 0.02 (0)	0.39b ± 0.01 (0)	0.40b ± 0.03 (0)	7.22a ± 0.04 (0)	6.00b ± 0.01 (0)	5.03c ± 0.01 (0)	5.00c ± 0.06 (0)
	20	15.00a ± 0.39 (6.56)	10.35b ± 1.25 (27.86)	10.15b ± 0.03 (25.89)	10.40b ± 0.32 (28.79)	0.39a ± 0.01 (4.40)	0.29b ± 0.06 (17.94)	0.30b ± 0.02 (23.61)	0.30b ± 0.05 (23.59)	6.80a ± 0.11 (5.84)	4.19b ± 0.33 (30.20)	3.80b ± 0.12 (24.33)	3.73b ± 0.23 (25.28)
*A. conyzoides*	40	14.32a ± 0.44 (10.83)	8.10b ± 0.63 (43.54)	6.76c ± 0.01 (60.61)	7.29bc ± 0.14 (50.09)	0.38a ± 0.03 (7.50)	0.21b ± 0.01 (39.15)	0.20b ± 0.05 (48.42)	0.21b ± 0.02 (47.54)	6.55a ± 0.01 (9.27)	2.75b ± 0.54 (54.08)	2.26b ± 0.11 (55.00)	2.24b ± 0.17 (55.09)
	60	12.57a ± 0.06 (21.73)	3.93b ± 0.19 (72.61)	3.34c ± 0.44 (75.62)	3.09c ± 0.12 (78.85)	0.32a ± 0.02 (20.38)	0.14bc ± 0.01 (59.54)	0.15b ± 0.07 (62.36)	0.12c ± 0.05 (69.37)	5.73a ± 0.14 (20.66)	1.27b ± 0.07 (78.81)	1.09bc ± 0.06 (78.22)	1.05c ± 0.01 (78.94)
	0	48.71a ± 0.29 (0)	45.53b ± 0.29 (0)	45.95b ± 0.68 (0)	45.45b ± 0.04 (0)	0.53a ± 0.01 (0)	0.58ab ± 0.02 (0)	0.57b ± 0.03 (0)	0.61c ± 0.04 (0)	14.80a ± 0.02 (0)	13.47bc ± 0.01 (0)	13.17c ± 0.05 (0)	14.08b ± 0.58 (0)
	20	47.88a ± 0.12 (1.70)	43.23b ± 0.52 (5.04)	43.33b ± 0.42 (5.70)	42.67b ± 0.06 (6.11)	0.52b ± 0.01 (1.57)	0.56b ± 0.02 (4.72)	0.54ab ± 1 (4.92)	0.57a ± 0.02 (6.36)	14.65a ± 0.16 (1.02)	12.35b ± 0.02 (8.37)	12.10b ± 0.10 (8.14)	12.78b ± 0.91 (9.22)
Weedy rice	40	46.78a ± 0.20 (3.93)	40.95b ± 0.13 (10.05)	39.07c ± 0.11 (14.96)	38.10d ± 0.05 (16.16)	0.51b ± 0.01 (3.29)	0.54a ± 0.01 (7.03)	0.51b ± 0.02 (9.97)	0.55a ± 0.05 (9.03)	14.23a ± 0.01 (3.84)	11.36b ± 0.02 (15.79)	11.20b ± 0.09 (15.00)	11.42b ± 1.26 (18.90)
	60	44.89a ± 0.23 (7.82)	32.84b ± 0.10 (27.87)	33.08b ± 0.69 (28.00)	32.56b ± 0.50 (28.37)	0.49a ± 0.04 (6.96)	0.45b ± 0.01 (22.20)	0.44b ± 0.05 (23.28)	0.50a ± 0.01 (18.07)	13.50a ± 0.50 (6.96)	9.63b ± 0.48 (28.53)	19.14b ± 0.25 (30.63)	9.67b ± 0.59 (31.32)
	0	46.84a ± 0.54 (0)	42.35b ± 0.10 (0)	42.23b ± 1.40 (0)	43.14b ± 0.08 (0)	0.41a ± 0.04 (0)	0.43a ± 0.04 (0)	0.38a ± 0.01 (0)	0.40a ± 0.02 (0)	13.09a ± 0.14 (0)	11.22b ± 0.07 (0)	11.22b ± 0.07 (0)	11.29b ± 0.01 (0)
	20	45.87a ± 0.10 (2.05)	38.72b ± 0.34 (8.57)	37.47c ± 0.30 (11.27)	38.39b ± 0.56 (11.01)	0.40a ± 0.03 (1.77)	0.41a ± 0.05 (4.87)	0.36a ± 0.02 (6.21)	0.37a ± 0.01 (8.51)	12.78a ± 0.14 (2.36)	10.01b ± 0.74 (10.74)	9.81b ± 0.62 (12.58)	9.74b ± 0.62 (13.73)
*C. iria*	40	44.24a ± 0.45 (5.54)	36.25b ± 0.70 (14.41)	34.75c ± 0.19 (17.72)	35.79b ± 0.44 (17.04)	0.39a ± 0.04 (3.50)	0.39a ± 0.01 (9.58)	0.33a ± 0.02 (13.38)	0.34a ± 0.03 (15.00)	12.23a ± 0.88 (96.55)	9.21b ± 0.50 (17.87)	8.96b ± 0.46 (20.08)	8.99b ± 1.10 (20.37)
	60	41.90a ± 0.54 (10.54)	28.23b ± 1.12 (33.33)	26.90b ± 0.10 (36.31)	27.47b ± 0.40 (36.33)	0.38a ± 0.10 (6.34)	0.32a ± 0.02 (25.45)	0.28a ± 0.03 (27.70)	0.29a ± 0.01 (28.94)	11.62A ± 0.01 (11.25)	7.32b ± 0.22 (34.69)	7.04b ± 0.03 (37.18)	6.95b ± 0.48 (38.48)

Data are expressed as means ± SD. Values with the same letters in the row among the hours after spray for each test weed are not significantly different at *p* > 0.05. Values inside the parenthesis are inhibition percentages relative to the control.

**Table 4:** Response of malondialdehyde and proline content of *A. conyzoides*, weedy rice and *C. iria* to a foliar spray of *P. hysterophorus* extract.

Test Plants	Dose (g L ^−1^)	Malondialdehyde Content (µmol g^−1^ FW)				Proline Content (µmol g^−1^ FW)			
		Hours after Spray				Hours after Spray			
		6	24	48	72	6	24	48	72
	0	1.90a ± 0.06	1.79a ± 0.14	1.71a ± 0.03	1.79a ± 0.14	5.06a ± 0.28	4.93a ± 0.14	5.01a ± 0.15	4.99a ± 0.08
	20	2.32a ± 0.09	2.58a ± 0.18	2.45a ± 0.06	2.62a ± 0.21	6.60b ± 0.23	7.03ab ± 0.14	7.40a ± 0.32	7.12a ± 0.10
*A. conyzoides*	40	2.71b ± 0.08	3.21a ± 0.22	3.14a ± 0.03	3.29a ± 0.24	7.83b ± 0.53	9.12a ± 0.29	9.41a ± 0.22	9.37a ± 0.06
	60	3.49b ± 0.06	4.52a ± 0.29	4.37a ± 0.05	4.67a ± 0.33	9.57b ± 0.69	12.14a ± 0.42	12.51a ± 0.46	12.58a ± 0.08
	0	2.80a ± 0.11	2.70ab ± 0.06	2.58b ± 0.09	2.56b ± 0.11	4.13a ± 0.24	3.75b ± 0.20	4.01ab ± 0.07	3.85ab ± 0.14
	20	2.90a ± 0.12	3.12a ± 0.11	3.00a ± 0.09	2.98a ± 0.12	4.31a ± 0.28	4.29a ± 0.24	4.67a ± 0.14	4.51a ± 0.28
Weedy rice	40	2.99b ± 0.08	3.39a ± 0.15	3.25ab ± 0.12	3.21ab ± 0.19	4.60b ± 0.21	4.73ab ± 0.28	5.14a ± 0.17	5.01ab ± 0.22
	60	3.28b ± 0.11	3.69a ± 0.06	3.59a ± 0.08	3.66a ± 0.12	4.99c ± 0.32	5.82b ± 0.38	6.53a ± 0.36	6.29ab ± 0.18
	0	2.74a ± 0.08	2.61ab0.15	2.45b ± 0.10	2.43b ± 0.04	3.76a ± 0.24	3.69a ± 0.13	3.98a ± 0.06	3.73a ± 0.10
	20	2.93a ± 0.02	3.12a ± 0.17	2.91a ± 0.13	2.90a ± 0.03	4.06b ± 0.34	4.35ab ± 0.25	4.78a ± 0.21	4.45ab ± 0.22
*C. iria*	40	3.06b ± 0.06	3.46a ± 0.20	3.21ab ± 0.11	3.22ab ± 0.07	4.33b ± 0.27	4.92a ± 0.16	5.29a ± 0.22	5.01a ± 0.20
	60	3.44b ± 0.10	3.82a ± 0.24	3.72ab ± 0.16	3.74ab ± 0.13	4.75c ± 0.26	6.04b ± 0.34	6.91a ± 0.15	6.49ab ± 0.21

Data are expressed as means ± SD. Values with the same letters in the row for each test weed among the hours after spray are not significantly different at *p* > 0.05.

**Table 5:** Response of superoxide dismutase, catalase and peroxidase activity of *A. conyzoides*, weedy rice and *C. iria* to a foliar spray of *P. hysterophorus* extract.

Test Plants	Dose (gL ^−1^)	Superoxide Dismutase (Unit g^−1^ FW)				Catalase (µmol g^−1^ FW)				Peroxidase (µmol g^−1^ FW)			
		Hours after Spray				Hours after Spray				Hours after Spray			
		6	24	48	72	6	24	48	72	6	24	48	72
	0	3.40a ± 0.07	3.35a ± 0.10	3.31a ± 0.04	3.29a ± 0.29	5.34a ± 0.20	5.15a ± 0.08	5.09a ± 0.11	5.04a ± 0.18	11.00c ± 1.93	12.77a ± 1.18	10.15a ± 0.92	12.72a ± 0.63
	20	4.11a ± 0.12	4.28a ± 0.15	4.25a ± 0.08	4.39a ± 0.38	6.00a ± 0.19	6.18a ± 0.03	6.26a ± 0.13	6.15a ± 0.20	12.38c ± 2.20	16.66ab ± 1.48	13.79bc ± 1.11	16.94a ± 0.72
*A. conyzoides*	40	4.85a ± 0.11	5.10a ± 0.12	5.07a ± 0.01	5.13a ± 0.46	6.48b ± 0.28	7.27a ± 0.19	7.35a ± 0.15	7.41a ± 0.29	14.15b ± 2.61	21.82a ± 1.93	17.40b ± 1.66	21.96a ± 0.87
	60	5.69b ± 0.10	6.89a ± 0.27	6.87a ± 0.06	7.11a ± 0.63	7.12b ± 0.36	8.18a ± 0.06	8.31a ± 0.17	8.28a ± 0.25	11.23c ± 3.08	27.63a ± 2.37	22.47b ± 1.93	28.22a ± 1.45
	0	4.01a ± 0.06	3.98a ± 0.04	3.79a ± 0.15	3.83a ± 0.17	5.88a ± 0.43	5.52a ± 0.25	5.86a ± 0.27	5..44a ± 0.37	19.74a ± 1.46	21.15a ± 1.08	21.71a ± 1.93	21.79a ± 1.60
	20	4.14a ± 0.07	4.31a ± 0.03	4.05a ± 0.15	4.07a ± 0.22	6.02a ± 0.38	5.80a ± 0.23	6.23a ± 0.31	5.81a ± 0.36	20.22b ± 1.57	23.94a ± 1.26	25.07a ± 1.91	25.15a ± 1.65
Weedy rice	40	4.27b ± 0.06	4.63a ± 0.07	4.42ab ± 0.12	4.45ab ± 0.17	6.16a ± 0.42	6.26a ± 0.28	6.70a ± 0.34	6.20a ± 0.34	20.86b ± 1.45	26.70a ± 1.34	28.45a ± 2.32	29.63a ± 1.82
	60	4.60b ± 0.12	5.22a ± 0.06	5.08a ± 0.18	5.17a ± 0.18	6.31a ± 0.40	6.77a ± 0.33	7.08a ± 0.30	6.69a ± 0.48	21.99b ± 1.46	32.06a ± 1.47	35.05a ± 3.12	36.09a ± 2.72
	0	3.99a ± 0.06	3.67c ± 0.06	3.85ab ± 0.13	3.73bc ± 0.03	6.02a ± 0.08	5.64ab ± 0.34	5.47ab ± 0.28	5.46b ± 0.29	17.48b ± 1.46	21.43a ± 0.92	19.96a ± 0.54	21.40a ± 0.92
	20	4.17ab ± 0.11	4.01b ± 0.05	4.23a ± 0.11	4.08ab ± 0.01	6.21a ± 0.12	6.11a ± 0.35	5.86a ± 0.30	6.01a ± 0.32	18.16b ± 1.40	24.87a ± 0.89	24.56a ± 0.71	26.31a ± 1.45
*C. iria*	40	4.31b ± 0.05	4.29b ± 0.06	4.53a ± 0.14	4.41ab ± 0.05	6.37a ± 0.05	6.49a ± 0.34	6.48a ± 0.28	6.60a ± 0.31	18.61c ± 1.46	28.25ab ± 0.93	27.21b ± 0.54	29.80a ± 1.21
	60	4.77c ± 0.06	5.05b ± 0.08	5.26a ± 0.16	5.21ab ± 0.02	6.58a ± 0.06	7.17a ± 0.43	7.01a ± 0.39	7.06a ± 0.37	19.74c ± 1.40	35.72ab ± 1.58	33.66b ± 0.70	36.46a ± 1.36

Data are expressed as means ± SD. Values with the same letters in the row among the hours after spray for each test weed are not significantly different at *p* > 0.05.

## Data Availability

The data presented in this study are available in the article.
